# The Caring Life Course Theory: Opening new frontiers in care—A cardiac rehabilitation example

**DOI:** 10.1111/jan.16312

**Published:** 2024-07-16

**Authors:** Maria Alejandra Pinero de Plaza, Claire Hutchinson, Alline Beleigoli, Matthew Tieu, Michael Lawless, Tiffany Conroy, Rebecca Feo, Robyn A. Clark, Hila Dafny, Penelope McMillan, Regina Allande‐Cussó, Alison A. Kitson

**Affiliations:** ^1^ Caring Futures Institute, College Nursing and Health Sciences Flinders University Adelaide South Australia Australia; ^2^ The Mparntwe Centre for Evidence in Health Flinders University: A JBI Centre of Excellence Alice Springs Northwest Territories Australia; ^3^ Adelaide Health Simulation The University of Adelaide Adelaide South Australia Australia; ^4^ Health Consumer Advocate with Lived Experience in Multimorbidity Disease Management Adelaide South Australia Australia; ^5^ Nursing Department, Nursing, Physiotherapy and Podiatry School University of Seville Seville Spain

**Keywords:** cardiac rehabilitation, caring life course theory, fundamental care, mixed‐methods, rural health

## Abstract

**Aim(s):**

To operationalize the Caring Life Course Theory (CLCT) as a framework for improving cardiac rehabilitation (CR) engagement and informing ways to address disparities in rural, low socio‐economic areas.

**Methods:**

A secondary analysis of data collected from 15 CR programmes to identify CR patterns through the CLCT lens using a mixed‐methods approach. All analytical processes were conducted in NVivo, coding qualitative data through thematic analysis based on CLCT constructs. Relationships among these constructs were quantitatively assessed using Jaccard coefficients and hierarchical clustering via dendrogram analysis to identify related clusters.

**Results:**

A strong interconnectedness among constructs: ‘care from others’, ‘capability’, ‘care network’ and ‘care provision’ (coefficient = 1) highlights their entangled crucial role in CR. However, significant conceptual disparities between ‘care biography’ and ‘fundamental care’ (coefficient = 0.4) and between ‘self‐care’ and ‘care biography’ (coefficient = 0.384615) indicate a need for more aligned and personalized care approaches within CR.

**Conclusion:**

The CLCT provides a comprehensive theoretical and practical framework to address disparities in CR, facilitating a personalized approach to enhance engagement in rural and underserved regions.

**Implications:**

Integrating CLCT into CR programme designs could effectively address participation challenges, demonstrating the theory's utility in developing targeted, accessible care interventions/solutions.

**Impact:**

- Explored the challenge of low CR engagement in rural, low socio‐economic settings.
- Uncovered care provision, transitions and individual care biographies' relevance for CR engagement.
- Demonstrated the potential of CLCT to inform/transform CR services for underserved populations, impacting practices and outcomes.

**Reporting Method:**

EQUATOR—MMR‐RHS.

**Patient Contribution:**

A consumer co‐researcher contributed to all study phases.

**Contribution?**

- Introduces Caring Life Course Theory to enhance cardiac rehabilitation engagement and nursing research and practice.
- Highlights the effectiveness of mixed‐method approaches in understanding complex care patterns.
- Encourages patient and public involvement in research for greater impact and relevance.

## 1 INTRODUCTION

Cardiac rehabilitation (CR) emerges as a critical component in the recovery journey of individuals with heart conditions, encompassing a holistic blend of medical, educational and behavioural interventions to facilitate optimal health outcomes (Beleigoli, Foote, et al., 2024). Despite its evident benefits, engagement and completion rates for CR are notably low (Beleigoli, Dafny, et al., 2024; Beleigoli, Foote, et al., 2024; Resurrección et al., 2019). Research identifies over 60 barriers to participation and persistence in CR programmes, from personal and clinical to logistical challenges, including psychological stress, lack of physician endorsement and geographical inaccessibility (Beleigoli, Foote, et al., 2023, 2024; Resurrección et al., 2019). These obstacles are further compounded for socially disadvantaged groups, underscoring the global urgency for customized intervention strategies (Beleigoli, Dafny, et al., 2024; Beleigoli, Foote, et al., 2023, 2024; Pinero de Plaza, Beleigoli, et al., 2024; Resurrección et al., 2019).

In Australia, the dichotomy between rural and urban, coupled with socio‐economic disparities, significantly hinders CR participation, thereby negatively affecting cardiovascular health (Beleigoli, Foote, et al., 2024). Astonishingly, studies found that only 11.2% of over 16,000 eligible individuals completed CR, despite a referral rate of 44.3% (Beleigoli, Dafny, et al., 2024; Beleigoli, Foote, et al., 2024). Identified barriers to completion included living alone and comorbid conditions such as diabetes and depression. Although telehealth programmes can facilitate improved access to care, they highlight the critical need for personalized mental health support, customized exercise regimens and peer support to address the unique needs of participants, particularly in rural and remote locations with low socio‐economic status (Beleigoli, Dafny, et al., 2024). These findings call for an urgent re‐evaluation of CR delivery, advocating for a person‐centred approach, primarily through telehealth, to bridge the gap in participation and outcomes for these vulnerable groups of the population (Beleigoli, Dafny, et al., 2024).

**FIGURE 1:**
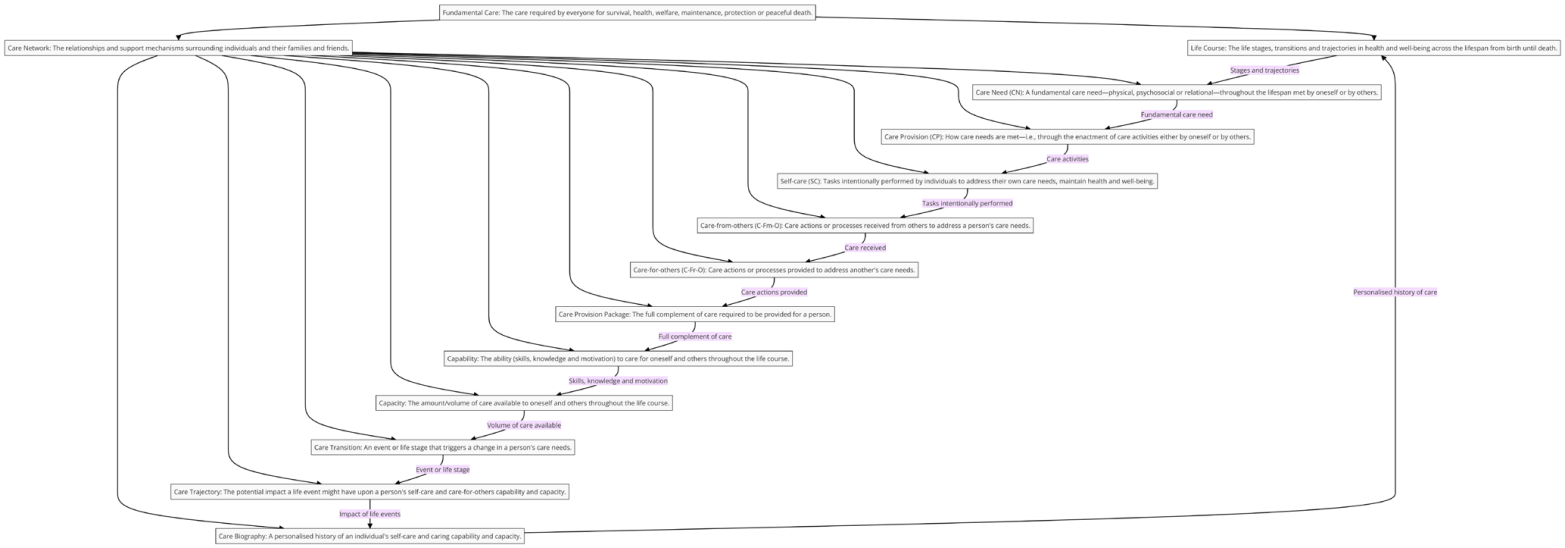
Our interpretation of the Caring Life Course Theory.

An emerging approach that may inform ways to bridge the gap in participation and outcomes in CR is the Caring Life Course Theory (CLCT), a multidisciplinary framework of 14 theoretical constructs (in Table 1) (Kitson et al., 2022). These constructs interact dynamically to define and meet an individual's needs in a contextually sensitive, care‐focused and adaptable manner (Kitson et al., 2022). To illustrate the constructs, we have depicted our interpretation of the CLCT in Figure 1, providing a comprehensive overview of its dynamic and workable assumptions and facilitating the comprehension of the theory's relevance for this care study.

**TABLE 1:** Caring Life Course Theory constructs (Kitson et al., 2022).

Fundamental care	The care required by everyone for survival, health, welfare, maintenance, protection or peaceful death, regardless of the presence or type of clinical condition or the setting in which care is taking place
Life course	The life stages, transitions and trajectories in health and well‐being across the lifespan from birth until death
Care network	The relationships and support mechanisms surrounding individuals and their families and friends
Care need (CN)	A fundamental care need—physical, psychosocial or relational—throughout the lifespan met by oneself or by others
Care provision (CP)	How care needs are met—that is, through the enactment of care activities either by oneself or by others
Self‐care (SC)	Tasks intentionally performed by individuals to address their own care needs, maintain health and well‐being, prevent and manage illness and attain specific goals
Care‐from‐others (C‐Fm‐O)	Care actions or processes received from others to address a person's care needs
Care‐for‐others (C‐Fr‐O)	Care actions or processes provided to address another's care needs
Care provision package	The full complement of care required to be provided for a person, made up of a combination of self‐care and care from informal, formal or professionals carers
Capability	The ability (skills, knowledge and motivation) to care for oneself and others throughout the life course
Capacity	The amount/volume of care available to oneself and others throughout the life course
Care transition	An event or life stage that triggers a change in a person's care needs
Care trajectory	The potential impact a life event might have upon a person's self‐care and care‐for‐others capability and capacity
Care biography	A personalized history of an individual's self‐care and caring capability and capacity and their understanding of the care they have and should receive from other people

The CLCT emphasizes the importance of comprehensively understanding and addressing an individual's fundamental care needs throughout life (Kitson et al., 2022). It recognizes the value of the social, economic and environmental determinants that shape health and care trajectories—a critical consideration for nurses and other health professionals in rural and underserved areas where resources are often limited for CR services (Beleigoli, Foote, et al., 2024; Kitson et al., 2022). The CLCT highlights the importance of early intervention, leveraging formal and informal social support networks (or ‘care networks’), and understanding the complex relationships between various factors at individual, relational and societal levels to enhance healthcare delivery and address individuals' complex needs effectively in such contexts (Kitson et al., 2022). Recognizing the critical role of high‐quality fundamental care and associated constructs in upholding health and preventing illness, the CLCT has the potential to empower clinicians and care providers to deliver enhanced services (Kitson et al., 2022).

**FIGURE 2:**
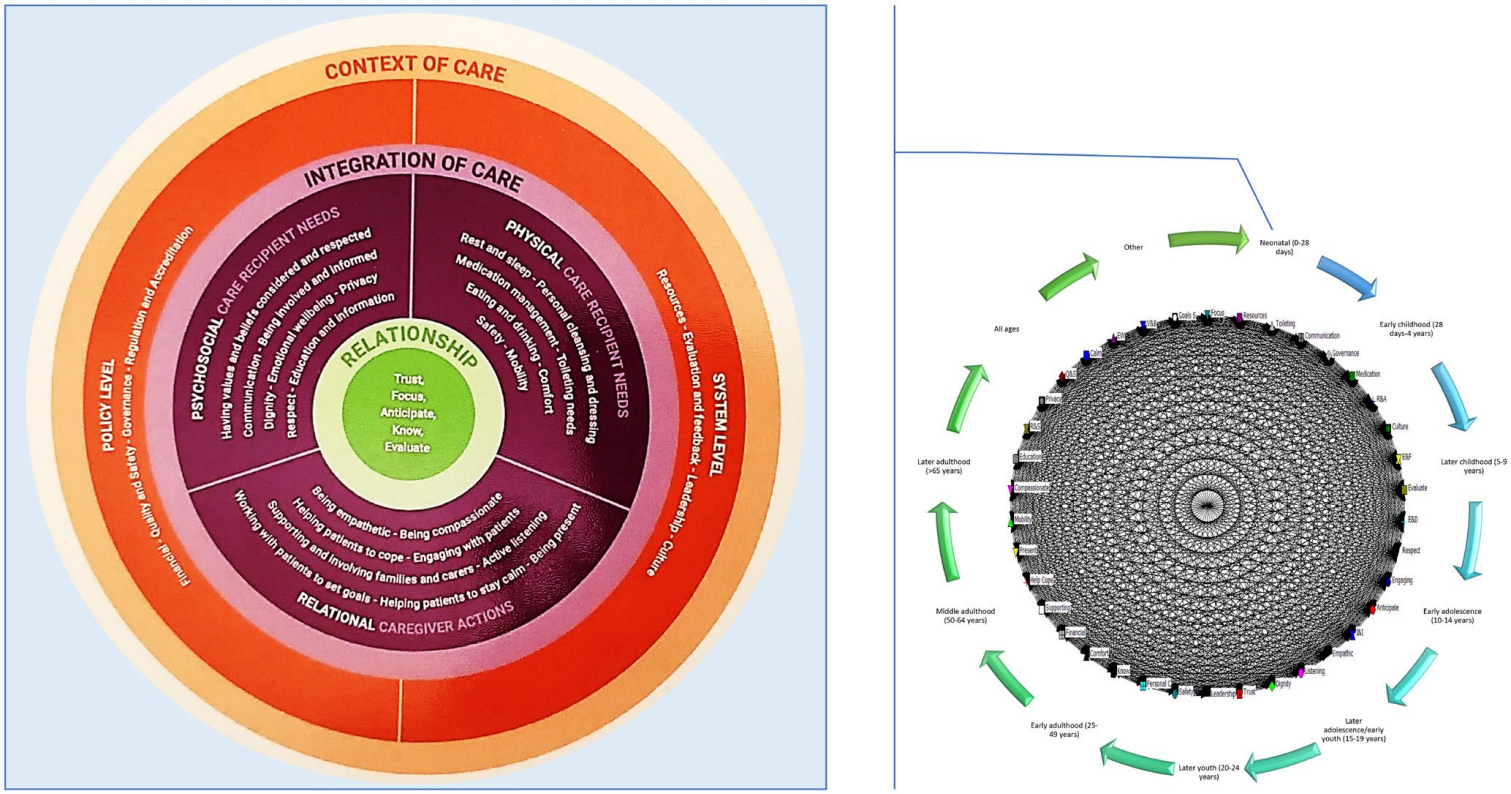
The fundamentals of care framework (top left) (Feo et al., 2018) and a network illustration of its components interacting across the life course (bottom right) (Pinero de Plaza, 2023a, 2023b).

In the CLCT, care constructs relate, adapt and reconfigure, ideally, in a combined and networking manner, based on a person's context, health status and circumstances where they come into play (Kitson et al., 2022; Pinero de Plaza, 2023a). Notably, within the CLCT (Kitson et al., 2022), the ‘fundamental care’ construct underscores the essential care requirements universal to all individuals for maintaining health, well‐being and survival, as per Figure 2 (Feo et al., 2018; Kitson et al., 2022; Lawless et al., 2023; Pinero de Plaza, 2023a).

**FIGURE 3:**
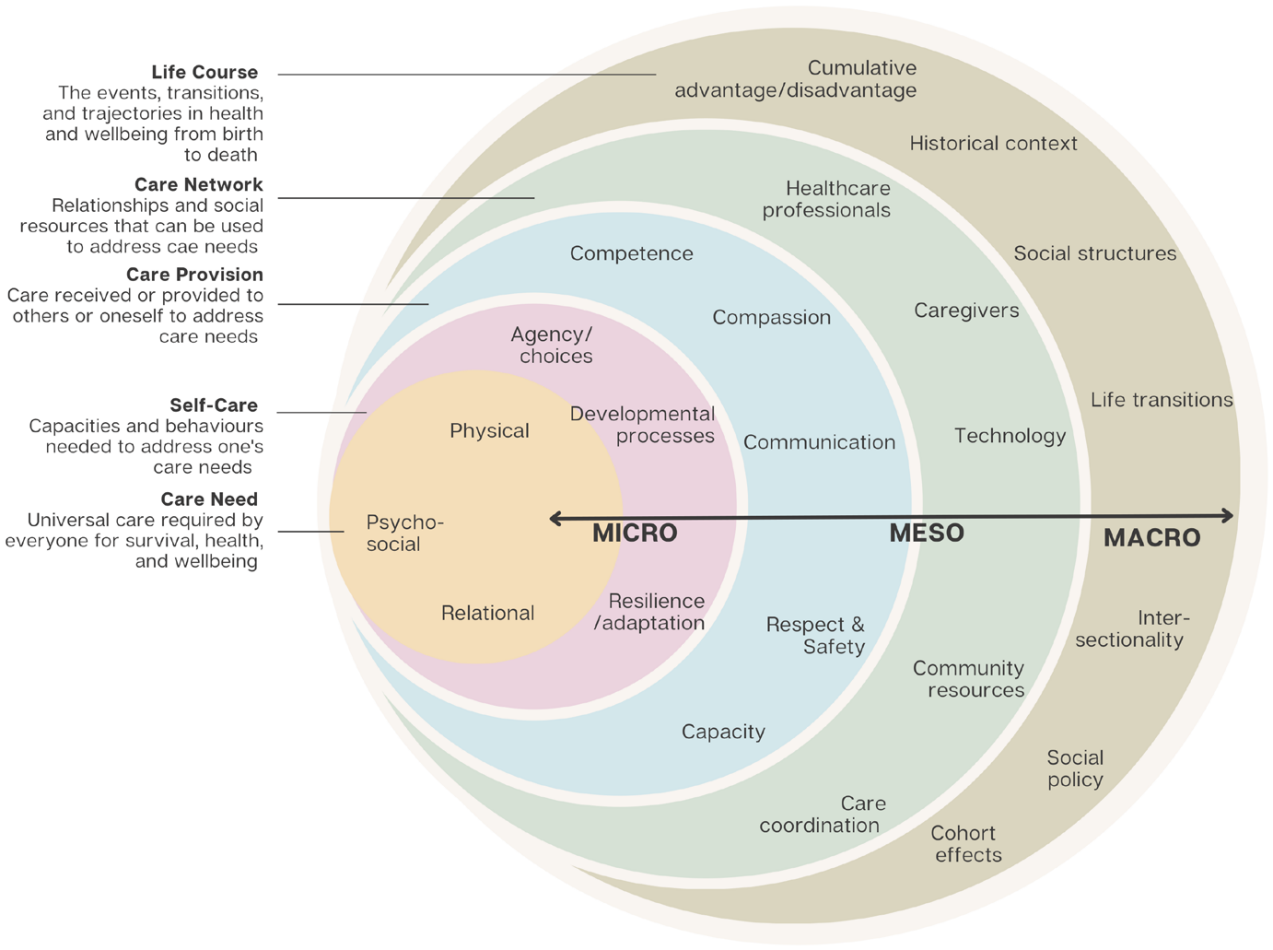
An overview of the Caring Life Course Theory at the micro, meso and macro levels (Lawless, 2023).

The CLCT has the potential to map, hypothesize and assess care dynamics comprehensively, as shown in Figures 1 and 2. Figures 1, 2, 3 illustrate the dynamic constructs of the CLCT and the elements of the fundamental care framework (Conroy et al., 2023; Pinero de Plaza, 2024; Pinero de Plaza, Conroy, et al., 2021; Pinero de Plaza, Yadav, & Kitson, 2023). These visuals aim to demonstrate how using precise and evidence‐based comprehensive language of care can enhance the understanding and assessment of its delivery and impact within complex health and social systems (Kitson et al., 2022; Tieu et al., 2022, 2023). Figure 3 highlights the importance of cohesive ‘self‐care’ and ‘care networks’ strategies, which integrate formal and informal care through various means such as natural, social, familial or technological (Kitson et al., 2022; Lawless et al., 2023; Pinero de Plaza, 2023a). This underscores the theory's potential in identifying optimal patient‐centred care across the healthcare system's micro, meso and macro levels, encompassing fundamental care and extending beyond (Lawless, 2023; Mudd et al., 2020; Pinero de Plaza, 2024; Pinero de Plaza, Conroy, et al., 2021; Pinero de Plaza, Yadav, & Kitson, 2023).

## 2 BACKGROUND

In this study, we leverage the CLCT proposed by Kitson et al. (2022) as a foundational framework to understand CR uptake and engagement; this theory affords a comprehensive systemic viewpoint (refer to Figures 1, 2, 3), which could enable the integration and identification of novel and systemic care strategies for CR. The impetus for adopting the CLCT framework stems from the rapid advancements in healthcare technologies and methodologies, including cutting‐edge telehealth, monitoring devices and innovative nurse‐led and behavioural strategies (Beleigoli, Dafny, et al., 2024; Beleigoli, Foote, et al., 2023, 2024; Bulamu et al., 2023; Bulto et al., 2023; Dafny et al., 2023; Gebremichael et al., 2022; Kitson et al., 2022; Mosleh et al., 2014; Suebkinorn et al., 2023). Despite the proliferation of these advancements, there is an emerging necessity to systematically evaluate the care potential of these developments to revolutionize CR services and care practice, especially for populations within rural and remote locations with low socio‐economic status, and to identify areas for CR engagement and completion enhancement (Beleigoli, Dafny, et al., 2024).

To understand the role of care from a broader perspective, it is essential to examine its meaning, components and patterns, considering their effects at the individual, organizational and systemic levels of healthcare systems. This endeavour marks a novel approach that goes beyond understanding the barriers and facilitators of CR. This exploration is crucial for identifying care pathways that could guide the development of strategies to enhance CR engagement and completion, addressing disparities and ensuring equitable healthcare outcomes and nursing approaches (Beleigoli, Dafny, et al., 2024; Beleigoli, Foote, et al., 2023, 2024; Bulamu et al., 2023; Bulto et al., 2023; Dafny et al., 2023; Gebremichael et al., 2022; Kitson et al., 2022; Suebkinorn et al., 2023). By operationalizing the CLCT as our guiding framework, we aim to refine and expand our current understanding of CR engagement and the role of care within it.

Therefore, this study thoroughly examines the hierarchical relationships among the CLCT constructs across various face‐to‐face and telehealth‐based CR programmes (Beleigoli, Dafny, et al., 2024; Beleigoli, Hutchinson, et al., 2023). These CR programmes have barriers and enablers identified via interviews and focus groups, as reported elsewhere (Beleigoli, Foote, et al., 2024); yet they have not incorporated the CLCT's systematic perspective. Therefore, this study seeks to fill this gap, proposing the generation of a CLCT‐based care blueprint as a method to inform and enhance strategic interventions for improving CR engagement and completion via an emerging and advanced understanding of care and nursing practices (Kitson et al., 2022).

## 3 DATA SOURCES

### 3.1 Method and study design

**FIGURE 4:**
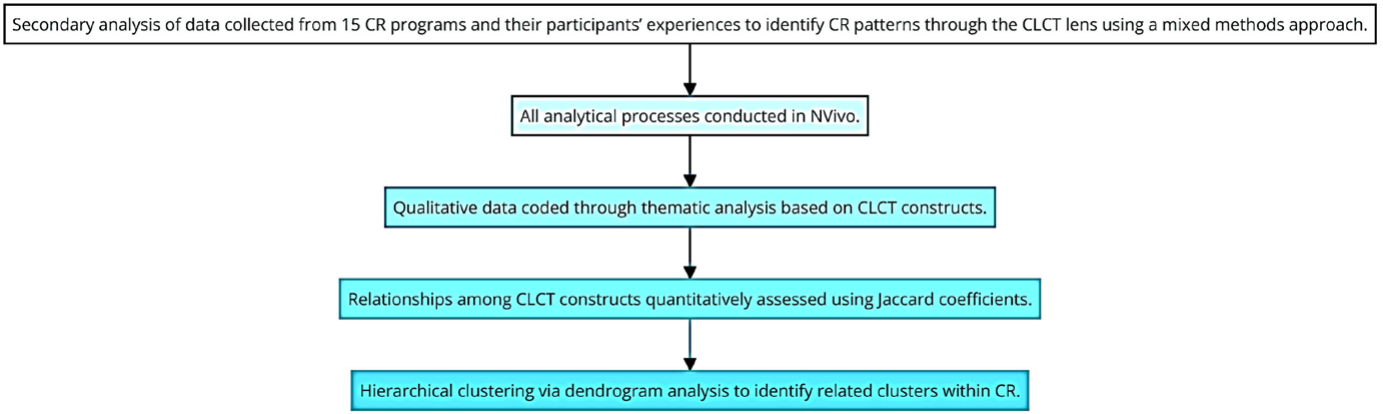
Mixed‐methods—QUAL → QUANT emphasis.

The primary study conducted by Beleigoli, Dafny, et al. (2024) used a mixed‐method design and exploratory sequential design (Beleigoli, Dafny, et al., 2024). Based on that work, this secondary analysis uses data from the primary study (Beleigoli, Dafny, et al., 2024), its 15 public CR programmes and their participants' experiences, to identify CR patterns through the CLCT lens using a mixed‐methods approach. All analytical processes were conducted in NVivo, coding qualitative data through thematic analysis based on CLCT constructs (coding example provided in supplementary material). Relationships among these constructs were quantitatively assessed using Jaccard coefficients and hierarchical clustering via dendrogram analysis to identify how such constructs gather into related clusters within CR, as presented in Figure 4.

Figure 4 presents a design that leverages methods to deliver an evidence‐based blueprint for enhancing CR engagement and completion by identifying patterns and gaps in care, potentially informing the creation of a promising pathway for developing tailored strategies to augment appropriate CR engagement and completion according to a CLCT perspective (Kitson et al., 2022) and Figure 1.

### 3.2 Setting and population

Data collection for the primary study occurred between 20 October 2022 and 25 November 2022, across rural and remote areas in South Australia by Beleigoli, Dafny, et al. (2024). Data were collected from both patients and clinicians. Inclusion criteria for patients were aged ≥18 years, referred to a CR programme in rural South Australia, residing in an area of low socio‐economic status defined by the lowest two deciles of the Index of Relative Social Advantage and Disadvantage (IRSAD) of the Australian Bureau of Statistics ((ABS) ABoS, 2016). Cognitive impairment (Mini‐mental short scale <20) was an exclusion criterion. Patients were predominantly male, aged 47–88 years, representing all six rural local health network regions, marital statuses, living arrangements, educational backgrounds and employment statuses; the participants engaged in cardiac rehabilitation for various heart conditions and predominantly for myocardial infarction as reported elsewhere (Beleigoli, Dafny, et al., 2024). The inclusion criteria for clinicians' participants comprised being a CR clinician of any discipline, mainly including cardiac rehabilitation nurses, especially females, coordinating services across any of the six regional (rural) local health networks in South Australia regardless of their years of experience—the time in the role was not an exclusion criterion (Beleigoli, Dafny, et al., 2024).

### 3.3 Recruitment and data collection

Within the primary study (Beleigoli, Dafny, et al., 2024), eligible patients were pre‐screened by the CR nurse and recruited by a researcher via telephone. The research team contacted eligible clinicians via email. Qualitative data were collected through interviews (4 with patients and 5 with clinicians) and focus groups (*n* = 24 patients). Interviews and focus groups were facilitated by three data collectors who explored cardiac rehabilitation experiences, focusing on patient journeys, clinicians' roles and programme enhancements for rural settings using the experience‐based co‐design framework (Boyd et al., 2010). In total, 28 patients and 6 clinicians participated in the primary study (Beleigoli, Dafny, et al., 2024). Audio recordings were transcribed verbatim and anonymized (Beleigoli, Dafny, et al., 2024).

### 3.4 Data analysis

As presented in Figure 4, to reveal clusters and patterns reflecting real‐world experiences associated with CR through the lens of the CLCT (Kitson et al., 2022), the qualitative data from the primary study (Beleigoli, Dafny, et al., 2024) were coded independently for this secondary analysis by co‐authors MAPP and CH (examples of such coding approaches are provided as supplementary material). Using NVivo software, they identified meaning units in the transcripts and coded them to the CLCT constructs, per the definitions and assumptions in Figure 1. The codes were then subject to Jaccard coefficient analysis. Jaccard coefficient analysis was chosen for the investigation because it is a robust method for identifying and analysing patterns of multimorbid health conditions in healthcare (Ng et al., 2018). It has previously been used to explore research topics and trends in nursing‐related communication in intensive care units (Son et al., 2018) and for a topic analysis on social media in breast cancer (Tapi Nzali et al., 2017).

NVivo software also enabled the identification of construct similarity distance via a dendrogram analysis to reveal hierarchical clustering among the CLCT‐coded constructs. The whole process behind this secondary analysis required over ten 1‐h sessions, in which a team of nine researchers from diverse fields (nursing, medicine, implementation science, knowledge translation, psychology, philosophy, public health, health promotion and a consumer advocate) analysed the hierarchical clustering results and their practical implications for enhancing engagement and participation in future CR projects and their nursing implications.

### 3.5 Ethical considerations

The study received approval from the Southern Adelaide Clinical Human Research Ethics Committee (LNR/22/SAC/71), Australia.

## 4 OVERVIEW OF THE ISSUE

Given that the CLCT (Kitson et al., 2022) has not previously been applied as a methodological lens in CR research, especially in rural and low socio‐economic statute areas; there is a significant gap in our understanding of how its constructs, in Figure 1, can illuminate the barriers to CR participation and completion. Without the prior application of CLCT in this context, we do not know if or how ‘fundamental care’ requirements are addressed within CR, and we do not understand how the ‘life course’ of individuals in these settings affects their CR engagement and how it has been reflected in their feedback. The role of ‘care networks’ in supporting or hindering CR participation remains unexplored, and the intricacies of meeting ‘care needs’ and supporting ‘care provision’ in such contexts are not well‐documented using the CLCT (Kitson et al., 2022).

Similarly, the potential impact of fostering ‘self‐care’, encouraging ‘care‐from others’, and facilitating ‘care‐for‐others’ within CR programmes is yet to be explored. The effectiveness of a comprehensive ‘care provision package’, the enhancement of ‘capability’ and ‘capacity’ for care, and the adaptation of CR programmes to suit ‘care transitions’ and ‘care trajectories’ have not been specifically examined using CLCT as a framework (Kitson et al., 2022). Moreover, the significance of considering ‘care biography’ in personalizing CR interventions to reflect individual health journeys and care experiences is an area ripe for investigation (Kitson et al., 2022). This lack of application signifies a vast, uncharted territory where the CLCT could revolutionize our approach to CR for underserved populations by offering detailed insights into the complex interplay of care needs, provisions and the broader care ecosystem. Therefore, to tackle the presented gaps, we operationalize the CLCT to address the following questions:

Research questions:

1. *What patterns emerge when interpreting cardiac rehabilitation within rural, low socio‐economic status contexts through the lens of the Caring Life Course Theory?*

Question for the discussion:

2 *How can these identified patterns guide future implementation projects to mitigate CR non‐participation or dropout within these communities?*

## 5 FINDINGS

1. Research question: *What patterns emerge when interpreting cardiac rehabilitation within rural, low socio‐economic status contexts through the lens of the Caring Life Course Theory?*

**FIGURE 5:**
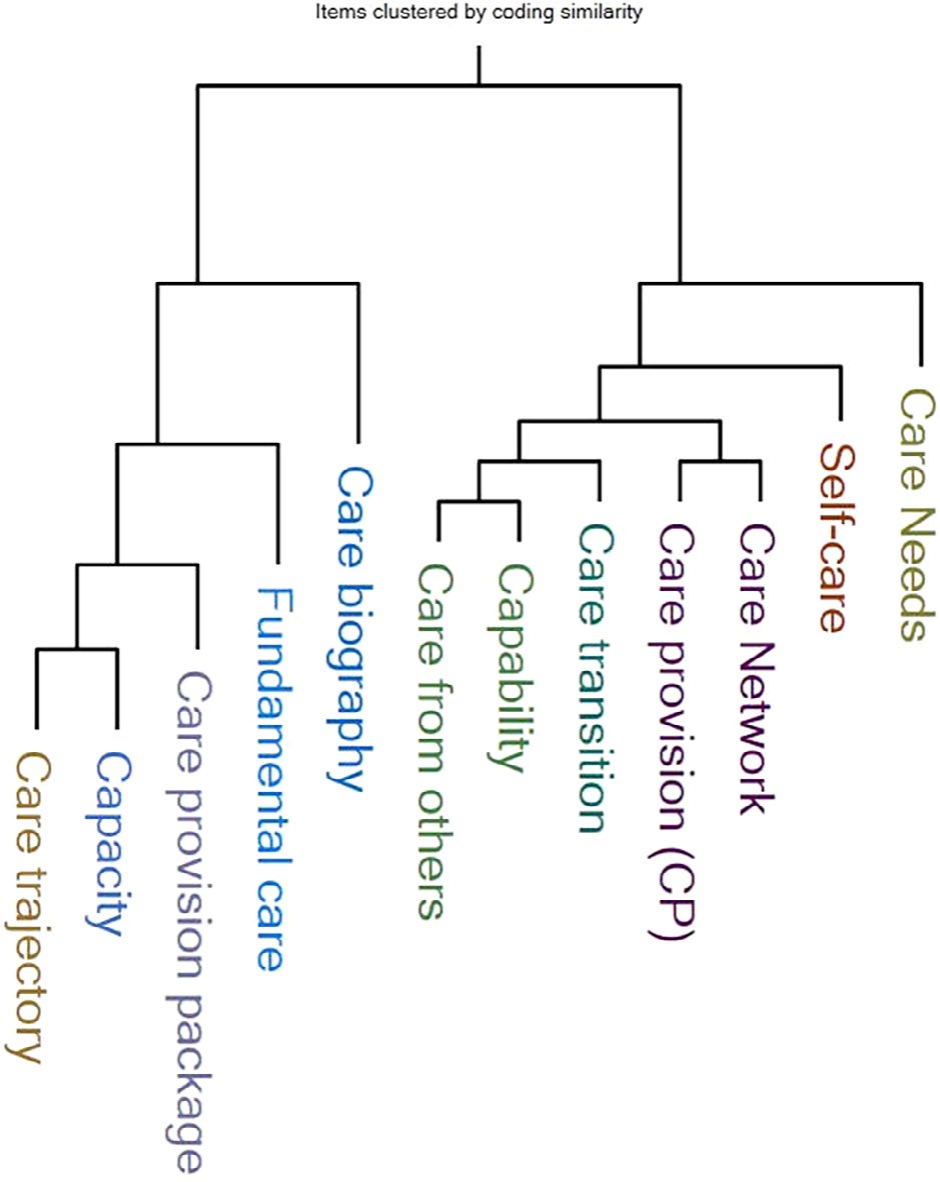
The foundational blueprint of CR within rural and remote locations with low socio‐economic status contexts (Jaccard's coefficients dendrogram).

The Jaccard coefficient results (fully reported in the supplementary material) underscore the interconnectedness of CLCT constructs like ‘care from others’, ‘capability’, ‘care network’ and ‘Care provision’ in supporting successful CR outcomes (Figure 5). These patterns suggest the essential integration of various care elements and the importance of considering the multifaceted nature of patient ‘care needs’ within CR programmes, as described below.

The dendrogram in Figure 5, created using NVivo based on Jaccard's coefficients, visually represents how the constructs of the CLCT are organized within rural and remote areas of low socio‐economic status. This dendrogram showcases the hierarchical relationships among CR patterns by closely grouping similar constructs on branches and separating distinct clusters into new branches. Constructs closely related and share similar characteristics are positioned near each other on the same branches, indicating strong similarity in how these constructs manifest within the population. In contrast, separating clusters into different branches highlights the differences in CR patterns, with less similar constructs diverging into separate branches, illustrating distinct characteristics or a unique blueprint of CR within the studied populations. Thus, the distance between constructs on branches quantifies the level of similarity: shorter distances within the same branch indicate a stronger relationship, whereas longer distances or separations into different branches reflect greater dissimilarity and distinct grouping of constructs.

The right branch of Figure 5 primarily encompasses constructs related to performing care and its transitions; thus, ‘care needs’ are displayed as supported by ‘self‐care’ and the latter supported by ‘care networks’, along with ‘care provision’ and ‘care transition’, showing the link between individual‐led care and the broader network involved in care delivery during the cardiac‐related care transition, which is sustained by the ability to care for oneself and others throughout the life course ‘capability’ and at the same level ‘care from others’.—conversely, the left branch of Figure 5 centres around individual ‘care biographies’ and ‘fundamental care’ provision. ‘Care biography’ encapsulates ‘fundamental care’, indicating that personalized biographical experiences are crucial for understanding fundamental care needs across life stages. Within such observations, ‘care provision packages’ are underpinned by ‘capacity’ and ‘care trajectory’, signifying that practical CR implementation connects to the planning of care provisions regarding the amount or volume of care available (‘capacity’), which relates to how the person receiving and/or delivering care reports on the life events impacting their CR trajectories (‘care trajectory’).

The Jaccard's coefficients calculations reinforce the dendrogram hierarchical placements by revealing the following:

*Perfect correspondence constructs (coefficient = 1)*: ‘care from others’, ‘capability’, ‘care network’ and ‘care provision’ exhibit perfect alignment, indicating substantial theoretical similarity and their crucial role in influencing patient outcomes within cardiac rehabilitation.

*Consistent similarities (high coefficients above 0.8)*: Constructs like ‘self‐care’, ‘care needs’, ‘care transition’, ‘care trajectory’ and ‘care provision package’ consistently exhibit high similarity coefficients, implying shared characteristics critical for comprehensive rehabilitation strategies.

*Moderate similarities (coefficients around 0.6–0.8)*: Constructs such as ‘capacity’, ‘fundamental care’ and ‘care biography’ demonstrate moderate similarity coefficients, highlighting notable relationships within these CLCT constructs.

*Disparities patterns (lower coefficients below 0.6)*: There are significant conceptual disparities between ‘care biography’ and ‘fundamental care’ (0.4), emphasizing the need to address conceptual divergences of reported experiences from the CLCT. Additionally, variations in constructs like ‘care provision package’, ‘care needs’ and ‘care trajectory’ below 0.6 suggest potential discrepancies in addressing fundamental care needs within the current CR approach. A notable dissimilarity between ‘self‐care’ and ‘care biography’ (0.384615) underscores the significant differences between an individual's self‐care practices and the biographical aspects shaping their care experiences within CR.

## 6 DISCUSSION

The CR blueprint derived from the dendrogram analysis underscores the importance of interconnected aspects such as ‘care needs’, ‘self‐care’, ‘care networks’, ‘care provision’ and ‘care transition’. These elements highlight the relationship between individual‐led care and their broader network during transitions in cardiac care. Understanding this multifaceted network is essential for effective self‐care in CR, necessitating the perspective of the social determinants of health—the conditions in which people are born, grow, live, work and age that shape health outcomes (Elam et al., 2022; Elias et al., 2019; National Academies of Sciences, Engineering, and Medicine, 2016; Pinero de Plaza, Beleigoli, et al., 2024; Willems et al., 2016). Critical factors influencing healthcare access, such as economic stability and education, significantly impact CR outcomes and extend beyond identified barriers and enablers, demonstrating that individuals with robust support networks (only 11.2%, as indicated by Beleigoli, Dafny, et al. (2024)) might navigate CR programmes more effectively than those with limited support (Beleigoli, Foote, et al., 2024; Carson, 2016; Kitson et al., 2022; Pinero de Plaza, 2023; Tieu et al., 2022).

Conversely, the left‐branch hierarchy within the dendrogram emphasizes central constructs within the CLCT, such as ‘care biographies’ and ‘fundamental care’, revealing how life experiences shape care needs across various life stages. This section displays a meaningful relationship between ‘care provision packages’, ‘capacity’ and ‘care trajectory’. It highlights the need to combine social policies and evidence‐based implementation in CR planning. The left branch shows necessary conceptual separation from the right, stressing the need for closer alignment between CR practices and the CLCT. Lower coefficients observed (in ‘care needs’, ‘care trajectory’, ‘care provision package’ and ‘capacity’) keep pointing to potential gaps in policymaking addressing fundamental care requirements. Thus, the left‐branch exploration delves into the meso to macro levels of the CLCT, encouraging consideration of intersectionality—the intricate interplay of social identities and systems, such as race, gender, class and sexuality, embedded within patient biographical knowledge (Beleigoli, Dafny, et al., 2024; Beleigoli, Foote, et al., 2023, 2024; Brown et al., 2016; Grønkjær et al., 2023; Hoff & Collinson, 2017; Holman & Walker, 2021; Rai et al., 2020; Resurrección et al., 2019).

The general gaps observed in this analysis are presented in Table 2 and discussed in detail afterwards.

**TABLE 2:** General gaps in current cardiac rehabilitation practices.

Construct	Theoretical integration	Practical implementation
Life course	The theory proposes a continuous and comprehensive view of care throughout an individual's life	In practice, care is often fragmented, with different stages and transitions not being cohesively managed or integrated, leading to disjointed care experiences
Care network	The theory emphasizes the importance of a supportive care network involving family and friends	Practically, these networks are often underutilized or not effectively integrated into formal care plans, missing out on the potential support they can offer
Care need (CN)	The theoretical framework considers a broad range of care needs, including physical, psychosocial and relational aspects	In real‐world scenarios, there is a tendency to focus primarily on physical needs, often neglecting care's psychosocial and relational dimensions
Care‐from‐others (C‐Fm‐O)	The theory acknowledges the importance of care received from others in meeting an individual's needs	In practice, the contributions of informal caregivers or the lack thereof, are often not formally recognized or integrated into the overall care strategy, leading to gaps in support and care
Care‐for‐others (C‐Fo‐O)	The theoretical model recognizes the actions and processes involved in providing care to others	Practically, there are challenges in documenting and supporting those who provide care, especially in informal settings. This can result in inadequate resources and support for caregivers and receivers

2. Discussion research question: *How can the identified patterns guide future implementation projects to mitigate CR non‐participation or dropout within rural, low socio‐economic communities?*

**FIGURE 6:**
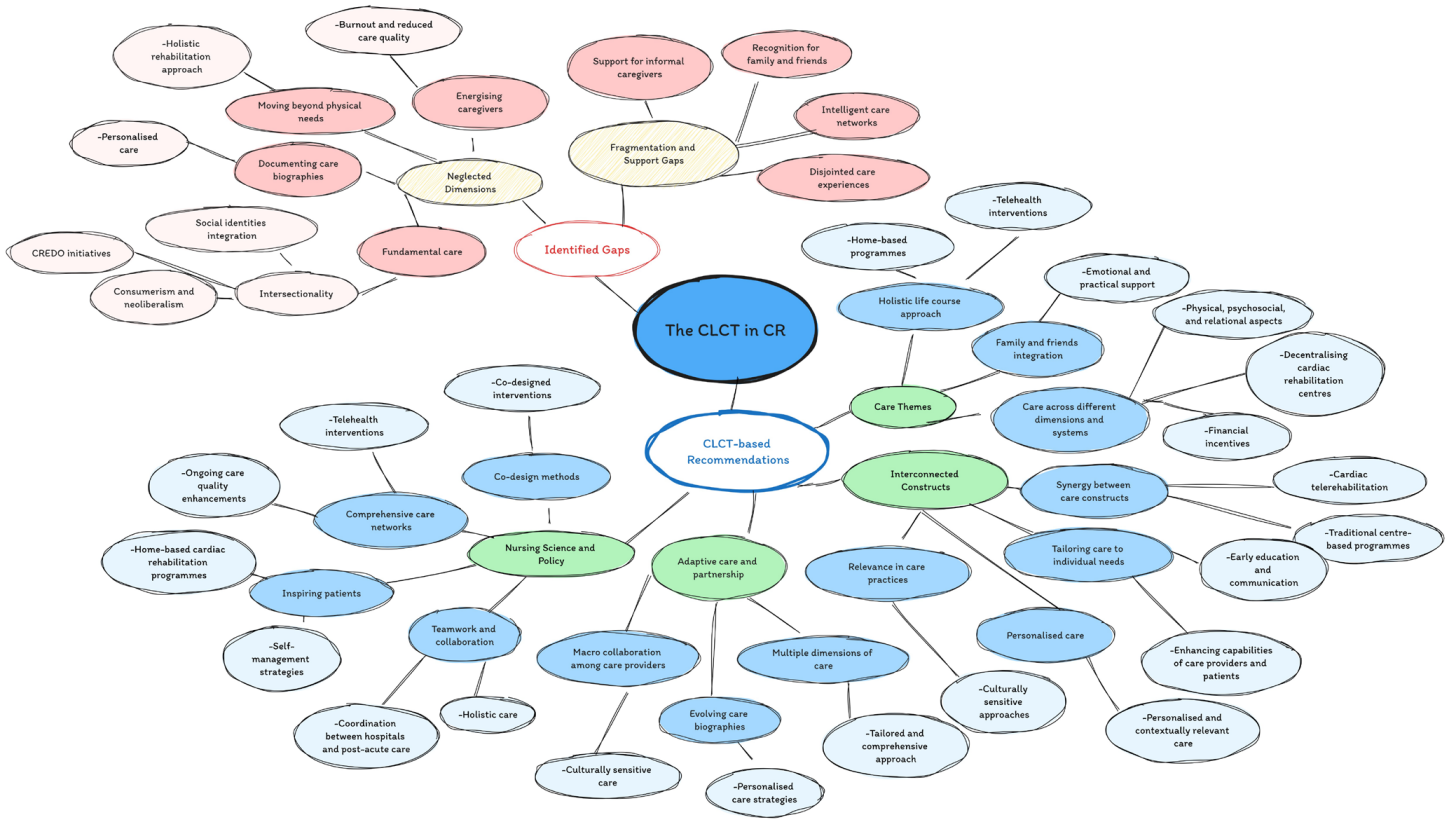
An analysis of gaps and recommendations for cardiac rehabilitation engagement considering the identified CLCT blueprint and current literature.

The identified gaps and patterns can guide future implementation projects through CLCT‐based recommendations, as illustrated in Figure 6 and supported by relevant literature.

Figure 6 presents a mind map using our CLCT findings to inform ways to bridge existing CR practice gaps. It aims to foster precise, compassionate, inclusive and resource‐efficient ways to improve CR, considering its stakeholders globally. It was created based on the quantifiable aspects of this study but informed by its qualitative assessment of CR (secondary coding analysis, as illustrated in the supplementary material); its ideas are discussed in light of the published global knowledge in CR and associated fields to highlight the profoundly interrelated aspects of the CLCT in CR and their complex relationships.

### 6.1 Identified gaps

#### 6.1.1 Fragmentation and support gaps

##### 6.1.1.1 Disjointed care experiences

Care experiences are often disjointed due to a lack of integration across different stages of CR. The absence of a seamless transition between care phases leads to fragmented support and coordination. More research is needed so that technologies, like artificial intelligence (AI), can be used to improve fragmentation aspects of CR (*New ways to solve complex problems and PROLIFERATE*, 2022). Factors around enrolment and participation may take priority as automatic physician referral systems have been shown to significantly increase enrolment rates, particularly following procedures like coronary artery bypass graft surgery (Smith et al., 2006). Using current technologies for a better institutionalized, physician‐endorsed system for CR could help address needs across the life course; perhaps other initiatives, such as linking primary care services with CR through nurse‐led prevention clinics, could be considered to enhance adherence and better CR patient outcomes (Goldman & Harte, 2020).

##### 6.1.1.2 Intelligent care networks

Supportive care networks, which include family and friends, are often not fully leveraged in the care process. This underutilization means that potential sources of practical knowledge are not effectively integrated into formal care plans; therefore, current models ignore real care capacity as understood within the CLCT. AI could support these processes; for example, the involvement of family and friends is vital for cardiac event survivors, as they require comprehensive discharge planning and rehabilitation services; so, new studies are looking at advanced ways to assess such family and expert networks to go beyond capturing patient‐reported measures and patient‐reported outcomes and model cardiac optimization strategies for better care (Pinero de Plaza, Gebremichael, et al., 2023; Pinero de Plaza, Lambrakis, et al., 2023; Sawyer et al., 2016). So, finding new ways of including family and other experts in CR needs research and testing of intelligent network approaches for peer support and local couches.  This may involve spouses as they are found crucial for CR but often overlooked (McGee, 1994). Intelligent care networks could optimize post‐myocardial infarction recovery by promoting positive health beliefs and understanding of physical activity regimens (Birtwistle et al., 2021) via better CR stakeholder understanding. Therefore, leveraging today's advancements may facilitate such new possibilities.

##### 6.1.1.3 Recognition for family and friends

There is a lack of formal recognition and integration of informal caregivers within the care strategy. This gap leaves informal caregivers unsupported and unacknowledged, creating significant burdens and gaps in the care continuum. Peer support and group training during the rehabilitation process have been identified as factors that can lead to better rehabilitation outcomes for geriatric patients (Tijsen et al., 2023). Moreover, compliance with rehabilitation programmes among patients with conditions such as schizophrenia and stroke is crucial to prevent negative impacts on both patients and caregivers, highlighting the need for tailored support and interventions (Anh et al., 2021).

##### 6.1.1.4 Support for informal caregivers

Informal caregivers face numerous challenges due to a lack of formal recognition, including managing disease, balancing care with personal life and dealing with mental stress (Pavlič et al., 2021). Despite their significant contributions, they often receive limited support from governments and healthcare professionals, leading to negative consequences for caregivers and care recipients (Ormel et al., 2017). Increased stress, burden and the risk of institutionalization for care recipients underscore the need for enhanced support and resources for caregivers (Laparidou et al., 2018).

#### 6.1.2 Neglected dimensions

##### 6.1.2.1 Energizing caregivers

Caregivers often feel unsupported and unrecognized by healthcare professionals, impacting their health and patient outcomes (Gusdal et al., 2016). Barriers to better CR include inadequate funding, lack of interdisciplinary training and inflexible interventions (Ratcliff et al., 2018). Addressing these issues to energize caregivers requires formal recognition, enhanced training and increased resources. Policies should fund effective technological solutions to support caregivers and improve health outcomes (Hassan, 2020). Coaching, informational resources, support groups, regular assessments and timely counselling can help caregivers cope (Gorawara‐Bhat et al., 2019; McHaro et al., 2023).

##### 6.1.2.2 Moving beyond physical needs

The emphasis in care often remains on physical needs, neglecting psychosocial and relational dimensions. This limited focus can result in inadequate care, failing to address the comprehensive needs of patients. Comprehensive cardiac rehabilitation is considered a cornerstone in the secondary prevention of cardiovascular disease. It is a tailored, multidisciplinary intervention that includes clinical evaluation, risk factor management, exercise training, dietary counselling and psychological and social support (Jegier et al., 2021). It aims to optimize pharmacotherapy, diagnose frailty, educate patients and families and monitor outcomes. CR programmes in Europe typically last 8–24 weeks or 3–4 weeks for inpatient programmes. Their teams should include doctors, physical therapists, nurses and technicians, with additional support from psychologists and dietitians in larger facilities. This underscores the significance of a holistic rehabilitation approach involving various aspects of patient care (Jegier et al., 2021). Yet, important longitudinal, meso and macro considerations on fundamental care and the CLCT are ignored.

##### 6.1.2.3 Documenting care biographies

The significance of integrating social identities in care across the life course is frequently highlighted but often poorly executed as patient‐centred approaches are considered flawed conceptually and practically (Tieu et al., 2022). Disparities in understanding and documenting patients' care biographies lead to gaps in personalized care. A lack of attention to individual histories and experiences can result in generic and less effective care strategies. Current care models often overlook fundamental care, leading to suboptimal outcomes, so more research and pilots are needed on nursing frameworks like the fundamentals of care framework and the CLCT (Conroy et al., 2023; Lawless, 2023; Mudd et al., 2020; Palese et al., 2020). Rehabilitation interventions focusing on targeted nursing models have been demonstrated to optimize cardiac function, reduce risk factors for heart failure, lower complication rates, enhance psychological well‐being and improve the overall quality of life for patients with acute myocardial infarction (Wang et al., 2022).

##### 6.1.2.4 Fundamental care

More research using the CLCT and fundamental care is needed because cardiac issues intersect with various social determinants and disparities. Integrating the CLCT with initiatives like the Coalition to Reduce Racial and Ethnic Disparities in Cardiovascular Disease Outcomes (CREDO) could better equip healthcare and CR professionals (Yancy et al., 2011). Researching intersectionality in CR is necessary as gender disparities in CR access show differences in psychological reactions and post‐cardiac event experiences between men and women (Angus et al., 2015; Jbilou et al., 2019). Studies have found that social isolation affects autonomic regulation of the heart and negative behaviours, highlighting the importance of social interactions in cardiovascular health (Barnard et al., 2015; Grippo et al., 2007). Discrepancies in outcomes after cardiac surgery are linked to social deprivation, stressing the need to address social determinants in cardiac care (Barnard et al., 2015). So, better CR requires considering fundamental care and the relationships supporting it, which requires moving away the emphasis from individualistic ideas of behavioural autonomy and personal responsibility because they are deeply rooted in consumerism and neoliberalism (Pinero de Plaza, Taghian, et al., 2021; Tieu et al., 2022).

### 6.2 CLCT‐based recommendations

#### 6.2.1 Care themes

##### 6.2.1.1 Holistic life course approach

The analysis emphasizes a holistic life course approach to care that ensures seamless transitions between different stages and phases of cardiac rehabilitation. This requires implementing alternative CR models, such as home‐based programmes and telehealth interventions (Epstein et al., 2021). Efforts should also focus on promoting referral for increasing participation and ensuring continuous care to reduce disparities (Dankner et al., 2014) and save resources (Thompson & Clark, 2009).

##### 6.2.1.2 Family and friends integration

The CLCT highlights the importance of integrating family and friends networks into the care process, recognizing their critical role in providing psychological, direct and tangible assistance (Beleigoli, Dafny, et al., 2024; Birtwistle et al., 2021; Luszczynska & Sutton, 2006). Studies have shown that the well‐being of spousal caregivers is positively linked to the regional availability of formal care, emphasizing the importance of formal care supply for care recipients and caregivers (Wagner & Brandt, 2017). Recognizing and supporting informal care networks through formal care provisions can lead to better care management and planning, ultimately improving the care system and covering individuals in need (Kontrimienė et al., 2019).

##### 6.2.1.3 Care across different dimensions and systems

Observed trends lead to considering a comprehensive range of care needs, including physical, psychosocial, and relational aspects, to deliver well‐rounded fundamental care, always pondering the influence of contextual factors (Kitson, 2018; Kitson et al., 2024). For example, considering the context dimension of fundamental care, decentralizing cardiac rehabilitation centres to socio‐economically deprived areas and using home‐based programmes may reduce transportation costs and improve equity in access to care (Ohm et al., 2023). This approach offers many benefits, including better service access, increased patient participation, improved clinical outcomes and cost‐effectiveness. It has been reported to enhance left ventricular ejection fraction, quality of life, physical fitness, recovery rates, self‐efficacy, physical activity, satisfaction, functional capacity, social support and haemodynamic parameters in cardiovascular disease patients (Nso et al., 2022).

Healthcare providers and policymakers should adopt decentralized rehabilitation models to improve cardiac rehabilitation services and recovery outcomes post‐cardiac events. Financial incentives have been suggested to increase participation among low‐socio‐economic status patients in CR (Gaalema et al., 2019), benefiting subpopulations that are underrepresented in such programmes (Dankner et al., 2014). Acknowledging the value of care received from others, including informal caregivers, and promoting their recognition and integration into formal care plans is critical. This care received from others is essential for enhancing patient outcomes and overall well‐being (Sangsaikaew et al., 2022; Verbakel, 2017). Thus, this study recognizes the various actions and processes involved, advocating for structured support for caregivers to enhance care quality (Jackson et al., 2024).

#### 6.2.2 Interconnected constructs

##### 6.2.2.1 Synergy between care constructs

Observed patterns imply that synergy between various care constructs is crucial and highlights the importance of coordinated efforts from multiple care providers. For instance, integrating CR with traditional centre‐based programmes has proven more effective and efficient in enhancing outcomes than centre‐based rehabilitation alone (Frederix et al., 2015). To that end, nurses are fundamental to generating the knowledge needed to enhance the professions around nurse‐led services. Therefore, observing, generating and training carers to create encouraging synergistic practices is not only important but necessary (Jackson et al., 2024).

##### 6.2.2.2 Tailoring care to individual needs

Enhancing the skills and knowledge of both care providers and patients to ensure that the care is specifically tailored to their needs is crucial to effectively customizing CR services. In low‐income regions, patients often lack awareness about CR during their hospital stay, underscoring the need for early education and effective communication (Beleigoli, Dafny, et al., 2024; Beleigoli, Foote, et al., 2024; Resurrección et al., 2019). Providing continuous nursing care based on structured diagnosis and treatment plans has been shown to enhance cardiac function, quality of life and adherence to rehabilitation among patients after percutaneous coronary intervention (Dai et al., 2022).

##### 6.2.2.3 Personalized care

Personalized and contextually relevant care involves incorporating personal contexts into care plans, ensuring they meet individual needs. This is relevant because implementing person‐centred care and customizing information enhances patient experiences and outcomes (Andersson et al., 2020; Wolf et al., 2019). Thus, care biographies can inform action/care plans and coping strategies, effectively promoting physical exercise in CR settings (Sniehotta et al., 2006). They can also help with telerehabilitation and home‐based CR programmes to improve accessibility and effectiveness in rural areas (Frederix et al., 2015; Takroni et al., 2022). Consequently, ensuring equitable, personalized access to CR requires the perspectives of the patient and their care networks as they capture and communicate the contextual limitations around establishing better referral procedures to address geographic, economic, and capacity constraints (Ngo‐Hamilton et al., 2024).

##### 6.2.2.4 Relevance in care practices

As older patients, women, individuals with low socio‐economic status and those with specific medical histories are less likely to participate in CR programmes (Kotseva et al., 2018), it is fundamental to create cultural relevance in care practices around CR and promote culturally sensitive approaches to meet diverse patient needs. For example, the primary study reported important differences in its sample socio‐demographic characteristics and, therefore, their perspectives: The participants are a diverse group predominantly composed of older males aged 47–88 years, fitting the typical demographic for CR (Beleigoli, Dafny, et al., 2024). Most participants were married, indicating varied levels of social support that could influence their rehabilitation journey. Employment statuses varied, reflecting different activity levels and availability for CR programmes. At the same time, educational backgrounds ranged from ‘year 11 or below’ to ‘graduate diploma/certificate’, emphasizing the necessity for CR programmes to address varying levels of health literacy (Beleigoli, Dafny, et al., 2024). Therefore, relevant, targeted solutions per each of these segments of the primary study population are necessary to improve engagement (Beleigoli, Dafny, et al., 2024; Beleigoli, Foote, et al., 2024; Pinero de Plaza, Yadav, & Kitson, 2023).

#### 6.2.3 Adaptive care and partnership

##### 6.2.3.1 Multiple dimensions of care

The CLCT integrates multiple dimensions of care to provide a comprehensive approach that simultaneously adapts to individual patient needs across their life; within those, tailored considerations are frequently demanded because they are crucial; studies have indicated that a lack of tailored and meaningful input according to peoples stages and transitions can decrease attendance rates among specific patient groups, such as communicated by studies on South Asian patients (Lotto et al., 2022).

##### 6.2.3.2 Evolving care biographies

CLCT encourages the inclusion of care biographies in care planning to ensure that care strategies are adapted to personal changes across the life course to remain effective. When care biographies are not included in CR, dropout rates are notably high; for example, despite the known benefits of rehabilitation and physical activity in alleviating depression, when young women with elevated depression and anxiety scores are not receiving integration of care and their feedback is not considered, they do not engage (Chandrasekhar et al., 2018). Moreover, characteristics like low 6‐min walk distance and muscle mass can predict dropout in cardiac rehabilitation; thus, the CLCT lens emphasizes the importance of comprehending the participants' profiles and their contexts, habitual behaviours and experiences (Pinero de Plaza, Taghian, et al., 2021) via the constant update of care biographies to reduce CR dropout rates (Choi et al., 2020) and the assessment of risk around non‐communicable diseases related to cardiac health (Pinero de Plaza, Taghian, et al., 2021).

##### 6.2.3.3 Macro collaboration among care providers

In this context, collaboration among care providers for adaptive and culturally sensitive care considers macro‐level aspects, such as socio‐economic drivers of disparity and behaviours. For instance, disparities exist in programme participation, with women with lower incomes and African American women being less likely to be referred and enrol in CR (Allen et al., 2004), and logically, patients in different socio‐economic subgroups feel excluded from CR (Pedersen et al., 2017); so, understandably, common reasons for declining centre‐based CR classes refer to accessibility issues, dislike of group settings and work or domestic commitments (Lee, 2016). Transdisciplinary teams and interprofessional learning and teaching strategies are recommended so these and other macro‐level obstacles, like economic conditions, are stopped from affecting CR engagement; for example, unemployment or job‐seeking, have been identified as a predictor of dropout in CR programmes (Pinero de Plaza, David, & Chipchase, 2022; Sloots et al., 2009).

#### 6.2.4 Nursing science and policy

##### 6.2.4.1 Teamwork and collaboration

This discussion of CR strategies frequently advocates for teamwork and collaboration in care from different angles. It aims to provide holistic care that addresses all aspects of patient well‐being. Therefore, collaboration is important; for example, coordinating CR care between hospitals and post‐acute care facilities remains challenging, impacting patient outcomes and access to rehabilitation services (Stoicea et al., 2017). Such a barrier, when seen via the CLCT, is augmented because of its interaction with ethnic minorities, low socio‐economic status patients and rural residents. They need new collaboration strategies between care providers and other care networks to change the current underutilization of CR (Beleigoli, Dafny, et al., 2024; Valencia et al., 2011), so existing methods and practices must be rethought to make a difference.

##### 6.2.4.2 Inspiring patients

The care view provided by the CLCT involves stakeholders in care planning to inspire patients to manage their care effectively. For example, home‐based CR programmes utilizing self‐management strategies with telephone contact or home visits have been identified as effective and valuable for rural and remote populations (Courtney‐Pratt et al., 2012). These programmes refer to strategies that foster a sense of agency and self‐efficacy, improve adherence to treatment plans, and promote long‐term health and well‐being. They involve nurses coaching patients about their conditions and self‐care techniques. They receive and operate the tools and resources needed to facilitate patients' effective coping with their care (Bulto et al., 2023; Gebremichael et al., 2023); yet, in those CR processes, it is fundamental, again, to acknowledge the complex interplay of social identities and systems for clinicians and patients, as their relationships are central to CR (Holman & Walker, 2021; Rai et al., 2020).

##### 6.2.4.3 Comprehensive care networks

Every type of care network is necessary to support CR (as per Figure 1), so ongoing enhancements in care quality are achievable by building and maintaining comprehensive patient care networks using relevant means. For instance, studies emphasize the crucial role of healthcare professionals in increasing referral and participation rates to outpatient CR, highlighting their valuable contributions in inpatient and home health settings (Arena et al., 2012). Still, further creative and updated means are important for adapting care network support. For example, telehealth interventions like weight management programmes and active video games have been explored and found to be feasible and effective (Barnason et al., 2019), as well as nurse‐led digital health strategies for cardiac health (Bulto et al., 2023; Sawa et al., 2022).

##### 6.2.4.4 Co‐design methods

For CR nurses and researchers, co‐design methods can help interaction with other professions as they encourage engagement with various stakeholders in the care process; for instance, connecting with patients to improve knowledge, co‐research and/or using participatory methods (Pinero de Plaza, Archibald, et al., 2024; Pinero de Plaza, Bester, et al., 2023; Pinero de Plaza, Gebremichael, et al., 2023) will promote awareness or change among relevant stakeholders (Archibald et al., 2021; Boyd et al., 2010; Pinero de Plaza, Kristina, et al., 2022). Therefore, the CLCT perspective advocates for continuous improvement in care strategies through co‐design, ensuring that care remains effective and responsive to patient needs (Pinero de Plaza, 2021; Pinero de Plaza, Beleigoli, et al., 2021, 2024). Such techniques are important to address wicked problems around multimorbidity, co‐morbidity, cultural barriers and low socio‐economic status (Birke et al., 2022; Tieu et al., 2023); thus, comprehending and including the viewpoints of health staff, community leaders, educators, policymakers and participants in rural and remote areas is critical to enhancing access and support for CR. Consequently, co‐design and system evaluation approaches are recommended (Field et al., 2021; Pinero de Plaza, Gebremichael, et al., 2023; Pinero de Plaza, Yadav, & Kitson, 2023; Tieu et al., 2023; Wiles et al., 2022).

#### 6.2.5 The role of CLCT and mixed‐method nursing approaches

The gap between ‘care biographies’ and ‘fundamental care’ in nursing practice highlights the need for clinicians to integrate these aspects into patient care plans, considering the patient's life course and biographical context to improve rehabilitation outcomes. Training, evaluation and research are necessary to understand, measure and apply these concepts, ensuring care is comprehensive, personalized and effective (Conroy et al., 2023; Pinero de Plaza, 2024; Pinero de Plaza, Conroy, et al., 2021; Pinero de Plaza, Yadav, & Kitson, 2023). Globally, changes in fundamental care emphasize addressing non‐nursing tasks, using positive language, accessing evidence and promoting interprofessional collaboration. This involves rethinking documentation and eliminating unproductive meetings to focus on high‐value fundamental care (Kitson et al., 2024). Integrating safety and quality practices into care services is crucial for enhancing CR outcomes and maintaining effective and affordable care systems (Kitson et al., 2024; Jackson et al., 2024).

Using the CLCT offers tangible means to bridge the divide between health and social care, moving beyond silos and geographical limitations. Leveraging formal care provision packages, digital communication tools and telehealth solutions, co‐designed interventions can bridge gaps between the CLCT and current CR practices (Beleigoli, Dafny, et al., 2024; Beleigoli, Foote, et al., 2023, 2024; Brown et al., 2016; Grønkjær et al., 2023; Hoff & Collinson, 2017; Resurrección et al., 2019). To that end, nursing research can inform CR programmes to identify needs, improve continuous care, build robust care networks and facilitate personalized, coordinated and community care through telehealth and nursing theories (Beleigoli, Foote, et al., 2024; Kitson et al., 2022) that also highlight and emphasize the monetary power of care (Jackson et al., 2024).

The presented mixed‐method nursing approach can encourage comprehensive evaluations of care models via the CLCT perspective to inform guidelines and resource allocation and elevate care standards (Barbabella et al., 2017; Beleigoli, Dafny, et al., 2024; Carson, 2016; Kitson et al., 2022; Pinero de Plaza, 2023a, 2023b; Pinero de Plaza, Beleigoli, et al., 2021, 2022; Pinero de Plaza, Conroy, et al., 2021; Pinero de Plaza, Yadav, & Kitson, 2023; WHO, 2018). Transnational calls from the nursing and multidisciplinary sectors are consistent with this study's recommendations and also refer to standardizing data sets and integrating patient‐generated outcome measures to encourage evidence‐based policies on fundamental care; such methods are being supported because of their potential to impact healthcare systems worldwide via a sounder understanding of care throughout patients' life journeys (Jackson et al., 2024; Kitson et al., 2022, 2024; Lawless, 2023; Pinero de Plaza, 2024).

### 6.3 Strengths and limitations

While our study offers valuable insights, it has limitations. Potential biases in self‐reported data and qualitative coding may affect our findings, and generalizing results to broader populations can be challenging. However, our method effectively captures detailed perspectives on CR programmes and should be adapted to rural communities globally. These findings are relevant to communities facing similar health challenges, such as poor health status, ageing populations and chronic diseases. Our CR recommendations may apply across diverse socio‐economic and cultural contexts. The CLCT framework shows promise but needs further testing for international applicability.

Integrating qualitative and quantitative data presents complexities, potentially limiting the direct translation of our findings without further localized research and co‐designed technological adaptations. Despite these limitations, we advocate for more research and application of fundamental care practices, the CLCT, and transdisciplinary care models. Thus, co‐design and evaluation strategies are recommended and necessary to improve cardiac rehabilitation engagement, technology implementation and overall health outcomes (Beleigoli, Hutchinson, et al., 2023; Pinero de Plaza, 2023; Pinero de Plaza, Archibald, et al., 2024; Pinero de Plaza, Yadav, & Kitson, 2023). Several studies support that an adaptable, patient‐centred approach can drive sustainable improvements in care delivery, fostering better engagement, health and well‐being globally (Athlin et al., 2018; Grønkjær et al., 2023; Kitson et al., 2019, 2024; Mudd et al., 2020, 2023; Muntlin et al., 2023; Nesbitt et al., 2023; Pinero de Plaza & Kitson, 2023; Voldbjerg et al., 2018).

## 7 CONCLUSION

Applying the Caring Life Course Theory to cardiac rehabilitation offers a novel way to understand and analyse care constructs, informing new individual, organizational and systemic strategies for personalized, resource‐saving, patient‐centred care. This approach highlights significant gaps between theoretical constructs and practical implementation, such as the fragmentation of care across the life course, the underutilization of care networks and the need for better integration of physical, psychosocial and relational care needs. Addressing the lack of inclusion and consideration of ‘care biography’ and ‘fundamental care’ data can help reduce non‐participation and dropout rates, ultimately enhancing cardiac rehabilitation engagement and outcomes. Those gaps underscore the importance of custom‐made care for underserved communities and the opportunity to incorporate culturally sensitive practices that address intersectionality and social determinants of health via new models. The operationalization of the Caring Life Course Theory offers detailed insights into care dynamics, advancing nursing science and improving care quality. It invites future programme development, focusing on longitudinal assessment of fundamental care and its implementation within healthcare systems globally.

## AUTHOR CONTRIBUTIONS

MAPP, CH, AB, and RC obtained the funding for this study. MAPP led all aspects of the project and created the design and methods. MAPP and CH coded the qualitative data. MAPP run the quantitative data analysis. MAPP, CH, AB, MT, ML, TC, RF, RC, PM, and RAC participated in 10 hours of sessions discussing design, findings, and implications. AB, RC, and AK provided senior co‐authorship via strategic advice and guidance. HD did the primary study qual analyses, with support from AB, MAPP, and CH, and provided important feedback for the interpretation of data and writing for this secondary analysis. PM collaborated in all study aspects, giving her consumer co‐researcher feedback and recommendations based on her lived experience of multiple disabling chronic illnesses and her voluntary advocacy work in ageing, carers, disability, chronic disease and ME/CFS. MAPP wrote the entire manuscript iteratively with the support of all authors. Every co‐author provided intellectual and writing improvements to create and approve the final version.

## FUNDING INFORMATION

This study was supported by a Flinders Foundation Health Seeding Grant for CRA4ALL (Cardiac Rehabilitation for All) and by the Country Heart Attack Prevention (CHAP) project, a peer‐reviewed National Health and Medical Research Council (NHMRC Partnership Grant GNT1169893). The salary of the lead author is covered by The College of Nursing and Health Sciences of Flinders University.

## CONFLICT OF INTEREST STATEMENT

The authors declared no conflicts of interest.

## PEER REVIEW

The peer review history for this article is available at https://www.webofscience.com/api/gateway/wos/peer‐review/10.1111/jan.16312.

## Supporting information


Figure S1.
Table S1.
Table S2.

## Data Availability

The data underlying this article cannot be shared publicly due to SA Health's policy for data linkage privacy. The data will be shared at a reasonable request by the corresponding author.
