# Supplementary material for: The Caring Life Course Theory: Opening new frontiers in care—A cardiac rehabilitation example
Source: J Adv Nurs. 2024 Jul 16;81(8):5163–80. doi: 10.1111/jan.16312 (PMC12271659; doi:10.1111/jan.16312)
Supplement: Supplementary file 1 — Figure S1. Table S1. Table S2. [file JAN-81-5163-s001.docx]

**Supplementary material**

Flowchart diagram and Table 1 examples of CLCT coding for this secondary analysis. The diagram illustrates the expanded CLCT constructs with summarised positive and negative examples from the primary study(3)’ data.


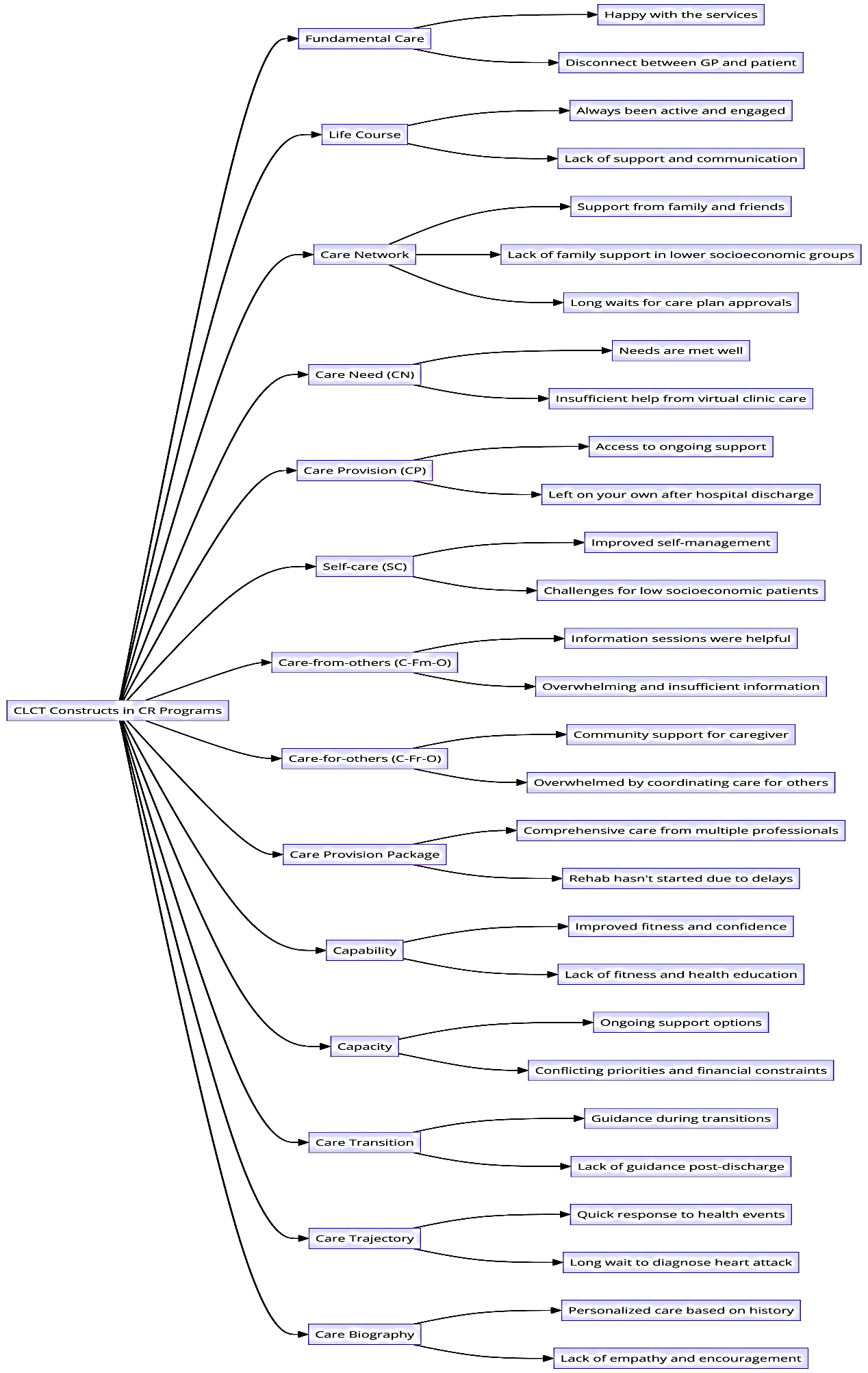


**Figure 1.** Flowchart diagram with CLCT constructs with summarised positive and negative examples.

**Table 1.** An example of the secondary analysis of the qualitative data coded thematically per CLCT constructs.

| Construct | Definition | Positive Quote | Negative Quote |
| --- | --- | --- | --- |
| Fundamental Care | The care required by everyone for survival, health, welfare, maintenance, protection or peaceful death, regardless of the presence or type of clinical condition or the setting in which care is taking place. | "I couldn't have been happier with the services. I really, I mean yes I'm a younger generation than all of you but I was actually quite happy with what was done and how it's run."​ | "There seems to be a big disconnect between my GP, who's my main carer, and even myself and them. I'm passing information on but, of course, it's my word. They're not going to take that. They're just typical medical profession. I was an ambo once. I know what it's like a little bit. It was different being on another foot. Very different from the wrong side of the bed. But the disconnect just seems to be there and they're not sharing information. That's really a bit of a gripe with me."​ |
| Life Course | The life stages, transitions and trajectories in health and well-being across the lifespan from birth until death. | "Looking back, I've always been active. When I was younger, I played sports regularly, and even now, I still make it a point to stay fit. Teaching gymnastics and helping out at the funeral parlor keep me busy and engaged. It's about setting a good example for the younger generation, showing them the importance of fitness and health throughout life. Despite some health scares, staying active has been key to my well-being" | "Life can be harsh. When I was young, I had big dreams and was full of energy. But as the years went by, things changed. I had to deal with a lot of setbacks, including health issues. It's hard to keep up the same level of activity and optimism. The lack of proper communication and support from healthcare providers made it even more challenging. Sometimes, it feels like nobody understands or cares about the struggles you face as you get older"​ |
| Care Network | The relationships and support mechanisms surrounding individuals and their families and friends. | "I was fortunate in one way. My son had a heart attack when he was 40. I had seen and helped in his recovery and seeing how he's recovered and what he can do now. So I sort of had that as a baseline. Knowing another couple of people locally who've had heart attacks and knowing how their rehabilitation has gone, I was reasonably relaxed that I was going to be okay"  "The support I had was a cardiac nurse used to phone me and I'd go through what I was doing, and she would just encourage me to keep on doing it. Phone calls about mental health support were very helpful. This particular lady, her first name Sherry, was brilliant in that area. She helped me out quite a bit actually just on a conversation as well as what else to do or what else to contact"​ | "I find the lower socioeconomic generally don't have a good family support. A lot of them have either just don't get along with their family or don't work or their kids they don't speak to them anymore or they're in trouble and I find a lot in our area. Probably a lack of family support. Just the lower socioeconomic people"  “I was only working a few weeks when I had a heart attack, and the care plan ran out. Those things are hard to do with the state of the health system at the moment because I can't get in to see the nurse until the 19th of December to start that care plan. This would've enabled me to go straight over to the physio today and just check up on the cardio side of things before I get in the gym. I had been doing the hydrotherapy and the pool and that was recommended. I don't get in the gym weights, and that's before the heart attack. I think it'd be good to go back to the same physio and talk about all this together. That's a long wait just for the nurse to okay a care plan. We don't even know if that's going to approve a care plan. These are long waits to get things like physio." |
| Care Need (CN) | A fundamental care need—physical, psychosocial or relational—throughout the lifespan met by oneself or by others. | "I feel like now that I've met Anne, my needs are much more being met than ever before. Puts a big load on the carer though, but the carer must be included in all instructions. Quite often the carer will remember things the victim doesn't mention" | "I got help through ICC And I didn't have half the stuff that he was talking about or dieticians and helping with exercise. I've had none of that help at all. Problems with my heart, I end up putting me through the VCC which is virtual clinic care, which now I do not every day, but no dieticians there's no helping with food or anything like that help with exercise programs, nothing just basic, just talking about what to do or how do I handle my heart failure" |
| Care Provision (CP) | How care needs are met—i.e., through the enactment of care activities either by oneself or by others. | "And then we offered a phase three maintenance exercise program for socially and disadvantaged people which again was at no cost and that was an 18-week program. That people could engage in. Now of course, COVID kind of shut all of that down. But having that access to that ongoing support at no cost is really important as well. So what we've done in the Barossa in the past is for people that we know are socially disadvantaged, financially disadvantaged, we would give them a 10-visit pass to the gym that we use for cardiac rehab so that they could continue on. And XXX, our personal trainer, who's just lovely, for a couple of people she's let them keep coming at no cost because she's just seen how much of a benefit it's been to their life." | "The reality is when you come out of the hospital in Adelaide, you're dumped on the footpath virtually and you're up to yourself how you get home. It's left to you. But I didn't find out one little thing that it would be a long ride. But I carry extra ambulance insurance pensioner thing, and if you come out of the hospital and you haven't got somebody to take you home or you can't fly. But if you get permission of the specialist that treated you, this ambulance thing that split in the pension package will take you from Adelaide. I know it's a long ride and it's free if you carry that insurance. It's just something I didn't know about until I rang up and queried what it was all about." |
| Self-care (SC) | Tasks intentionally performed by individuals to address their own care needs, maintain health and well-being, prevent and manage illness and attain specific goals. | "Did you feel like you were able to better self-manage having those kind of expert education from different health professionals? Oh absolutely. Because we're always learning. We always think we might know things and then you might read books and all that. It's having a look at documentation that heart book we've been talking about is excellent. It's obviously we've got the internet as well without information overload but then dealing with the specialist because there's always little bits and pieces that you can that once you listen to it and understand it can only improve your health moving forward. Even if it's just diet and things like that. Or maybe I am drinking or eating too much sugar or something you know? It just gives you a little wake-up call but at the same time you know it puts in your mind what you need to do to move forward and start living a bit more healthier." | "Do you think that patients from a low socioeconomic background would experience the same outcomes as somebody who wasn't necessarily from a low socioeconomic background? No, I don't think they do. Very few of them. And even I guess their ability to engage with general practice on an ongoing basis that's going to be beneficial. These are the people that drop off the radar that you might have an appointment booked but then can't make it because there's some other crisis. I kind of liken it to a train wreck really where these things are just happening all the time in their life that are just one crisis after another. And it's really often just about survival from day to day let alone thinking 10 or 15 years down the track where is their life going to be." |
| Care-from-others (C-Fm-O) | Care actions or processes received from others to address a person's care needs. | "The information session and they were brilliant. We've also had this a couple of years ago now. We had a chemist, a dietician, a doctor, and someone with heart problems. They were excellent. What was excellent too was the community within the group of suffering with all health conditions. You sort of one minute you find the next minute your life someone's just slammed against the wall and set your life on the line. It was all of a sudden you had to live a different way and do things differently. It's like, hang on, what happened? Then the emotional impact was something that each of the participants could also relate to. That was really helpful with each other." | "Well, I found that there's not a lot of time to speak with these people and they are very busy, but it's overwhelming as a patient and sometimes you're not sure whether you heard it. I'm sure there's information you didn't get because they're in and out in a brief moment, especially with the cardiologist. You can't talk to him when you're in the theater, obviously, you're out and then they're so busy. I saw her for like three minutes once in seven days. The information you get when you're in pain with painkillers, some of it just washes over and then later on you're trying to work it out yourself." |
| Care-for-others (C-Fr-O) | Care actions or processes provided to address another's care needs. | "I had to take care of my husband after his heart attack. It was tough, but we managed with the help of our community. Neighbours would drop by with meals and offer to drive him to his appointments. Our daughter also stepped in to help with household chores and ensure we had everything we needed. This support system made a significant difference in managing his recovery and easing the burden on me" | "It was really challenging having to coordinate all of my husband's medical appointments while managing the household and my own health issues. I often felt overwhelmed and exhausted, especially when there was no one else to step in and help. The healthcare system didn't provide much support in terms of managing these responsibilities, leaving me to figure out everything on my own" |
| Care Provision Package | The full complement of care required to be provided for a person, made up of a combination of self-care and care from informal, formal or professional carers. | "During his cardiac rehabilitation program he had a lot of health professionals care for him throughout. For example you have your cardiac nurse, physiotherapist, dietician, and so forth. On the actual phone hookup. Video hookup yes. Having access to those different professionals was very helpful because they are specialized in different areas, and having that comprehensive support made a significant difference in my recovery" | "My rehab hasn't actually started. It should be face to face. With the follow-up appointment with the cardio or with the physio, the nurse at Angus and the rehab nurse made the phone calls for me, and the hospital had put me in. They hadn't told me about this yet, so I'm assuming that only happened when the nurse called them. The date to see a cardiologist was going to be in February next year. She got back on the phone and changed all that, and I'll be seeing a different cardiologist in a few weeks, which is helpful. With the physio, I had been on a care plan relevant to the osteoarthritis conditions that had put me out of work and had helped me get physio and myself back to employment"​ |
| Capability | The ability (skills, knowledge and motivation) to care for oneself and others throughout the life course. | "They can do more than they previously could in terms of fitness. They are more confident in their management. Their emotional wellbeing normally scores improve. Their depression score generally improves, their fitness generally improves by doing the six-minute walk test. We can normally check on that or the sit-to-stand test depending on what we can put in. Again, they pick up things that they've previously given up. They feel more informed and confident about managing their heart health and action plans."  "I found that as I was breaking each goal, I was breaking down those barriers of being depressed. So, every time I hit a goal and not only hit it but exceeded it, I was getting that praise from Frank and from Annie asking how I was doing. They literally always ask, 'How is everybody doing? How are you all going?' And that's probably their best and most powerful question they can ask." | "No, I don't think people were honest because, look, uh, XXX, if you know me and you know other people, I don't look 70 because I don't drink and I don't smoke. Yeah. Whereas a lot of people, you know, I think in life it deals a bad card every now and again. Either they've lost a loved one or they've given up in life or they don't try, and you know, they can look a little bit different. Yeah. Than other people. And there was a lot of people there that basically couldn't get out of their chair at my age. A lot of it stems from when they're younger to teach 'em what to do. A lot of people do not know how to do fitness, and they don't even know. So it's a sad situation in life." |
| Capacity | The amount/volume of care available to oneself and others throughout the life course. | "And that’s where we’re really fortunate in a sense because we have a lot of options for ongoing support. We offered a phase three maintenance exercise program for socially and financially disadvantaged people at no cost. This was an 18-week program where people could continue to engage in physical activity and receive ongoing support. It’s been invaluable for many, allowing them to maintain the great choices they’ve made and continue their progress."  "I think one of the key outcomes is growth in their confidence to return to an active life. Whether that be returning to work or travel if they’re a retiree, gaining that confidence back that actually there is life after a cardiac event and I can be fit and well and I can do those things that I'd already planned before this unexpected thing happened to me and then my world was turned upside down." | "For some of them, you can't even get really to what you think is important for them to know because they can't afford their rent or their car's broken down again and they can't get to you. There are so many conflicting things happening in their life that prioritizing their health is not on their highest list. So some people that I guess the important thing is that there's no charge for our service. That is incredibly important." |
| Care transition | An event or life stage that triggers a change in a person's care needs. | "I think the telephone is good in the beginning; after that, face to face is essential. Initially, they say, 'You've got to walk half an hour,' but in my case, going to the hospital where they have the machine and someone telling you what to do is invaluable. It makes you feel confident and ensures you're doing it right." | "I think one discharge the week hospital and a week in the city with a friend in the third week going back to a rural area the only contact I could make was the desk at the cardio ward in the hospital. They are very busy. What was lacking is the little things that build your confidence like how far to walk and how much pain should you be in. It might be as simple as a training nurse with a checklist of questions provided by a cardiac but none of that was available. You're left on your own to wonder if you're traveling or not. Most of the time you don't feel like calling an ambulance because you're not quite there. Then your second guessing is this pain a serious one or not. Those early days were pretty scary going through that." |
| Care trajectory | The potential impact a life event might have upon a person's self-care and care-for-others capability and capacity. | "Well, I ended up in an accident and emergency one weekend Saturday. They gave me medication but there was no available bed in the hospital. So I was staying home after getting the medication with the information to attend the GP on Monday and be very sure I do attend the GP. I ended up back in there on Sunday, the next day, as nothing had changed. It was still fast. They started on medication, and they were going to admit me but there was no ambulance from Adelaide to come up. And then they found out there were no available beds in Adelaide in the cardiac units and there was no nursing staff available here. So again, I was sent home with the information to make sure to see my GP the next day, which I did do. Then I ended up having to drive myself down to see the specialist to get a scan, and then drive back home. Despite all the difficulties, I managed to get the care I needed." | "I was flying to Adelaide like well you were but there was nothing to show that I'd actually had a heart attack. So it wasn't until probably nearly eight months down the track that they did an MRI scan and found Yes I had paid one and then things started to click in. But I went through that six eight months without really knowing." |
| Care biography | A personalised history of an individual's self-care and caring capability and capacity and their understanding of the care they have and should receive from other people. | “In their initial assessment we try and do a little bit of a social history. If they're on a pension or if they're privately retired or if they're working, if they're renting. We do a really brief background history. If they've got good family support, we have a little tick box to see like if they've got good family support, if they're living alone after they, are they isolated are they vulnerable thing. We've got quite a few of those things. Already in our systems like the vulnerability checklists we've got isolating isolated risk and all those kind of things. We would have a bit of a gauge, but then again, it's not something that we probably would like triage them by or give them any different education. I suppose we could provide a bit more support if we felt that they needed it, but we wouldn't really change their program as such.” | “Everything saying to me, I have had to take the test myself to get to where I am and take responsibility for test. I see the cardiologist, I say, I'm on this program, I'm doing this. Oh yeah, that's good. There's no, oh what is that.... I mean I'm not putting them down. I mean he's getting the medication obviously this stuff. But is that lack of empathy and the lack of well that's good that you're doing that. Gives a bit of explanation. Ask me a couple of questions. Some interest at least. Don't just blanket Yes. And try and encourage you to do it because it's a great thing. These chances that seen people that only cause you know, I've lost 20 kilos since of staff. Nothing said about that. Cardiologist didn't see anything... The GP does need be clothes, which is always, but there is no encouragement I don't think from anyone there.... I think it's more, and I'm upset to people on the course.” |

**Table 2.** Jaccard Coefficient Results

| **Code A** | **Code B** | **Jaccard's coefficient** |
| --- | --- | --- |
| Care from others | Capability | 1 |
| Care Network | Capability | 1 |
| Care Network | Care from others | 1 |
| Care provision | Capability | 1 |
| Care provision | Care from others | 1 |
| Care provision | Care Network | 1 |
| Care transition | Capability | 1 |
| Care transition | Care from others | 1 |
| Care transition | Care Network | 1 |
| Care transition | Care provision | 1 |
| Self-care | Capability | 0.928571 |
| Self-care | Care from others | 0.928571 |
| Self-care | Care Network | 0.928571 |
| Self-care | Care provision | 0.928571 |
| Self-care | Care transition | 0.928571 |
| Care Needs | Capability | 0.857143 |
| Care Needs | Care from others | 0.857143 |
| Care Network | Care Needs | 0.857143 |
| Care provision | Care Needs | 0.857143 |
| Care transition | Care Needs | 0.857143 |
| Care trajectory | Capacity | 0.8 |
| Fundamental care | Care trajectory | 0.8 |
| Self-care | Care Needs | 0.785714 |
| Care provision package | Capacity | 0.777778 |
| Care trajectory | Care provision package | 0.777778 |
| Care provision package | Care biography | 0.714286 |
| Self-care | Capacity | 0.692308 |
| Capacity | Capability | 0.642857 |
| Care from others | Capacity | 0.642857 |
| Care Network | Capacity | 0.642857 |
| Care provision | Capacity | 0.642857 |
| Care trajectory | Capability | 0.642857 |
| Care trajectory | Care from others | 0.642857 |
| Care trajectory | Care Network | 0.642857 |
| Care trajectory | Care provision | 0.642857 |
| Care transition | Capacity | 0.642857 |
| Care transition | Care trajectory | 0.642857 |
| Fundamental care | Capability | 0.642857 |
| Fundamental care | Care from others | 0.642857 |
| Fundamental care | Care Network | 0.642857 |
| Fundamental care | Care provision | 0.642857 |
| Fundamental care | Care transition | 0.642857 |
| Fundamental care | Capacity | 0.636364 |
| Fundamental care | Care Needs | 0.615385 |
| Fundamental care | Care provision package | 0.6 |
| Self-care | Care trajectory | 0.571429 |
| Self-care | Fundamental care | 0.571429 |
| Care biography | Capacity | 0.555556 |
| Care trajectory | Care biography | 0.555556 |
| Self-care | Care provision package | 0.538462 |
| Care Needs | Capacity | 0.5 |
| Care provision package | Capability | 0.5 |
| Care provision package | Care from others | 0.5 |
| Care provision package | Care Network | 0.5 |
| Care provision package | Care provision | 0.5 |
| Care trajectory | Care Needs | 0.5 |
| Care transition | Care provision package | 0.5 |
| Fundamental care | Care biography | 0.4 |
| Self-care | Care biography | 0.384615 |
| Care biography | Capability | 0.357143 |
| Care from others | Care biography | 0.357143 |
| Care Network | Care biography | 0.357143 |
| Care provision | Care biography | 0.357143 |
| Care provision package | Care Needs | 0.357143 |
| Care transition | Care biography | 0.357143 |
| Care Needs | Care biography | 0.214286 |

Jaccard coefficient results were computed via NVivo to depict a dendrogram and care patterns.
